# Case of Strangulated Vermiform Appendix; Operation; Death; Remarks

**Published:** 1880-11

**Authors:** 


### Selections.

---

CASE OF STRANGULATED VERMIFORM APPENDIX; OPERATION; DEATH; REMARKS. (Under the care of Mr. PICK.)

The case in which the vermiform appendix of the cæcum had descended into the sac of a hernia and had there become so tightly strangulated as to produce very severe symptoms, necessitating operation and terminating in speedy death, appears to be worthy of being placed on record on account of its rarity.

Robert E., aged fifty-four, painter, was admitted into the hospital on February 21st. He stated that he had ruptured himself twelve years previously, but that his hernia, which he had always been able to return, had never given him any trouble nor prevented him following his occupation. He had therefore paid no attention to it, and had never worn a truss. Five days before admission his rupture came down, and he was unable to push it back again. Soon he began to suffer great pain in the part, and it became much swollen. His bowels had not acted since the descent, and he had been constantly sick, the vomit latterly having been of a very offensive nature. The day before admission a medical man made a prolonged attempt by taxis to reduce the hernia, but without success. The proceeding caused him great pain.

When admitted he was in a state of collapse. The surface of the body was cold and covered with a clammy sweat; the pulse was scarcely to be felt, and very quick; the tongue was coated with a white fur; the abdomen was tympanitic, but not rigid. In the right inguinal region and lower part of the abdomen, extending nearly to the level with the umbilicus, the tissues were swollen and œdematous, and the skin was red. Just below and internal to the abdominal ring was a tense, hard swelling, which occupied the upper part of the scrotum and extended up into the inguinal canal, along which considerable fullness and hardness could be felt. There was no impulse on coughing, and the patient did not complain of much pain during the examination.

He was at once placed under the influence of ether, and a vertical incision was made over the external abdominal ring. Upon cutting into the subcutaneous tissue a large quantity of serum escaped. The various coverings of the sac were then divided. They were much infiltrated, and adhered to each other. The sac, having been exposed, was opened, and a large quantity of thick fetid pus escaped. The sac wall was very much thickened, and was coated on its internal surface by flakes of lymph. After the fluid contents of the sac had been carefully sponged away, the sac was found to contain only the vermiform appendix. The portion of intestine was much thickened and congested, and, like the anterior of the sac, was covered with flaky lymph. The stricture, which was so tight that it was with difficulty divided, was situated at the neck of the sac. After the division of the stricture the finger could be passed freely up into the abdominal cavity. The vermiform appendix, which was not perforated, was now carefully cleansed, and returned just within the opening in the abdomen. As the sac was in a state of acute suppuration, it was dissected out from the tissues with which it was in contact and removed; the external wound was then closed. The operation was performed with strict antiseptic precautions. During the operation the patient was very faint and collapsed, and required brandy. He never rallied after his removal to bed, and died in about five hours.

*Necropsy.*—There was a wound some three inches long over the right inguinal canal, closed by silver suture. The wound being opened gave access to a small sac communicating directly with the peritoneal cavity by its neck, which was about one-third of an inch in diameter. At the upper part of the sac was the extremity of the appendix cæci, adhering by soft adhesions, which were easily broken down, to the lining membrane of the neck of the sac, which was continuous with the peritoneum. The rest of the appendix and the cæcum were close to the opening of the sac in the abdominal cavity. At the point of junction of the appendix to the cæcum was a ring of discoloration, and here the tube was compressed and slightly ulcerated. The appendix was thickened and covered by flakes of lymph, but otherwise appeared tolerably healthy. The peritoneum in the neighborhood of the sac was dark colored, showing a little soft lymph on its surface. There was no general peritonitis. The lungs were very emphysematous and congested. There was puriform fluid in the bronchi. The heart weighed ten ounces; it was uncontracted. There was a little partially decolorized clot in the right ventricle. The muscular tissue was very soft and friable, and had undergone considerable fatty change. The valves were natural. The liver weighed $3\frac{1}{2}$ lb.; it was soft and pale, and infiltrated with fat. The other organs were natural.

Mr. Pick remarked in connection with the above case that it was one of great rarity. He had never certainly seen a similar case. Nor did he even remember to have read of one where the vermiform appendix was strangulated. Of course cases where a portion of the bowel, probably cæcum, and the vermiform appendix together were found in a hernial sac were not uncommon. He had looked through the tables of operations for strangulated hernia in St. George's Hospital for the last twenty-eight years, and had not been able to find an instance of the same thing mentioned in these records, nor had he found the subject alluded to in any of the authors he had consulted. It seemed difficult to account for the way in which the vermiform process had become strangulated. How a tube of such small dimensions as the appendix should have been so tightly constricted in a canal which on previous occasions had allowed the passage of a much larger body, as a portion of the gut, or, at all events, omentum. For it seemed perfectly clear to his mind that the hernial sac must on a former occasion have contained either gut or omentum, for he could scarcely conceive how a soft, free, movable tube like the appendix could produce a hernia by forcing before it the peritoneum along the inguinal canal, though it was easy to understand how it might find its way into a hernial sac when it was once formed. He could come to no other conclusion than this, that the sac had been originally formed by the protrusion of a portion of intestine or omentum in the usual way, and into the sac thus formed the appendix has descended. It seemed strange under these circumstances that it should have become stangulated, and at the operation he had expected, when he saw what was in the sac, to find a small knuckle of intestine high up by the side of the appendix, but that this was not the case was abundantly proved by the post-mortem examination, which showed that the whole of the intestine was natural, except this little process, and that this had been tightly constricted was proved by the ring of ulceration which surrounded it, at the point of constriction, close to its junction with the cæcum. He believed that the strangulation must be explained by supposing that some inflammatory exudation had taken place in the tissues external to the neck of the sac, and that this was the cause of the stricture. From whatever cause it had arisen it was very tight, for it was with the greatest difficulty that the extremity of the hernia knife could be got beneath it to divide it.

Another point of interest was, he said, the acute symptoms which were produced. One would scarcely have imagined that constriction of the appendix would have produced such urgent symptoms, and such early vomiting, for it must be borne in mind that this was a very different thing to strangulation of a portion of the intestine, in which the perfect condition of the tube was essential to life. With regard to the appendix cæci it was not so; this tube might become obliterated without any absolute detriment to the patient, and Mr. Pick supposed that if the operation could be performed with safety it might be removed without in any way interfering with the natural functions of the individual. The symptoms of strangulation were due, no doubt, to arrested peristaltic action; but it was difficult to understand why this arrested peristaltic action should have taken place so suddenly from constriction of a small tube like the vermiform appendix, which was not essential to life. Mr. Pick further stated that at the time of the operation he was in considerable doubt as to how he should deal with the appendix; whether he should return it into the cavity of the abdomen, or whether, having freed the constriction, he should leave it in the sac. On the one hand, it seemed a hazardous proceeding to return it, actually inflamed as it was, and covered over by shaggy lymph, into the abdominal cavity; and on the other hand, if it were allowed to remain, it would undoubtedly contract adhesions to the tissues with which it was in contact, and would therefore always remain as an incurable hernia, and might possibly become again strangulated by the contraction of the tissue during cicatrization. Under these circumstances he decided upon pushing it back just within the cavity of the abdomen, and leaving it there in the expectation that it would contract adhesions to the peritoneum in the neighborhood of the abdominal ring. And that this did occur was shown by the post-mortem examination, when it was found that it was already adherent in this situation.—*London Lancet.*

